# A genealogical approach to academic success

**DOI:** 10.1371/journal.pone.0243913

**Published:** 2020-12-17

**Authors:** Mignon Wuestman, Koen Frenken, Iris Wanzenböck

**Affiliations:** Innovation Studies, Copernicus Institute of Sustainable Development, Utrecht University, Utrecht, The Netherlands; University of Minho, PORTUGAL

## Abstract

We analyse academic success using a genealogical approach to the careers of over 95,000 scientists in mathematics and associated fields in physics and chemistry. We look at the effect of Ph.D. supervisors (one’s mentors) on the number of Ph.D. students that one supervises later on (one’s mentees) as a measure of academic success. Supervisors generally provide important inputs in Ph.D. projects, which can have long-lasting effects on academic careers. Moreover, having multiple supervisors exposes one to a diversity of inputs. We show that Ph.D. students benefit from having multiple supervisors instead of a single one. The cognitive diversity of mentors has a subtler effect in that it increases both the likelihood of success (having many mentees later on) and failure (having no mentees at all later on). We understand the effect of diverse mentorship as a high-risk, high-gain strategy: the recombination of unrelated expertise often fails, but sometimes leads to true novelty.

## 1 Introduction

The question whether evolutionary theory can be applied to the social sciences is as old as evolutionary theory itself [1]. Analogous to biological evolution as a process of genetic reproduction, cultural evolution has been considered as a process in which particular items (such as knowledge, skills, practices and values) are being passed on across generations. Such cultural items are replicated in various social contexts, including through upraising in families, teaching in schools and training in firms [2].

The evolution of scientific knowledge is part of the process of cultural evolution and can thus be studied as such [3]. Scientific knowledge, skills and practices are passed on between generations of scientists primarily through training at universities. Its study can be done through a genealogical lens, as academic training is institutionalized in the form of Ph.D. supervision. Anyone who graduates has been mentored by one or more Ph.D. supervisors (*mentors*) and any graduate can in turn become a supervisor of one or multiple Ph.D. students (*mentees*) later on. Arguably, Ph.D. supervisors do not just train Ph.D. students in a particular knowledge domain, but also selectively pass on their own ideas and skills within this domain [4]. This implies that the evolution of scientific knowledge is also, in part, driven by genealogical dynamics: mentors with more mentees are able to pass on their knowledge at a higher rate than those with fewer mentees.

In previous studies, academic success has been related to characteristics of good mentors [5–8] and scientists’ personal relationship with their mentors [9, 10]. Genealogies of scientists have also been studied before to characterize scientific disciplines or institutes [11], develop indicators of field interdisciplinarity [11], and intellectual heritage [12]. Others have used genealogies to investigate to what extent mentees replicate their mentors’ career choices and success [5], and more specifically to analyse the extent to which Nobel laureates are part of the same mentoring communities [13].

Our study starts from the idea that recombination plays a key role in scientific advance. Analogous to the logic of genetic recombination driving biological evolution, we understand cultural innovation as stemming from new combinations of ideas and artefacts [14, 15]. In the context of science, Ph.D. students may experience a variety of influences, and combining these may help them to produce creative ideas. The potential for recombination can be traced back from the diversity of cultural influences that an individual inherits from its past mentors. Note that genealogical linkages between scientists in terms of mentorships are not only direct, but also indirect covering any number of past generations (mentors-of-mentors) [7]. The whole genealogical past of individuals may, in principle, affect their ability to produce new ideas, and, in this way, there future careers.

Genealogies can be modelled as networks. A genealogical network is a directed network that represents the channels of knowledge transmission from mentor to mentees, as well as–reversely–the opportunities for invention by mentees who combine mentors’ knowledge. Every scientist can be considered as a node in a directed network, where the in-degree stands for the number of mentors that a scientist has had as a mentee, and the out-degree stands for the number of mentees that a scientist mentored later on in the academic career. Studying mentor-mentee relationships is relevant, because the number of mentees of a mentor offers a way to understand academic success, one that reflects the selection passing on of knowledge to new generations [5, 7]. Indeed, having more mentees has been shown to be highly correlated with publication output and academic prestige [5]. In a genealogical approach to science, the number of Ph.D. students trained in the course of a career can thus be used as a measure of academic success. That is, in network terms, a mentor’s academic success can be expressed by a mentor’s out-degree. In this framework, one can analyze what network properties affect a mentor’s out-degree. We do so by analyzing the genealogical data of mentor-mentee relationships using a large-scale dataset of 95,502 scientists for the period after World War II.

The central question we aim to answer holds how a scientist’s mentors at the time of Ph.D. training affect future academic success. First, we investigate this question by looking whether one’s out-degree as indicator of success (number of mentees) is increased by one’s in-degree (number of mentors). Second, following the idea of recombination underlying success, we investigate how the genealogical diversity between one’s mentors affects one’s success later on. We hypothesize that having a greater ‘cognitive diversity’ of mentors will increase the likelihood of both success (having *many* mentees later on) and failure (having *no* mentees later on). As more diverse mentors provide mentees with more opportunities for creative recombination of knowledge, skills and practices, there is a higher chance that a mentee produces radically novel ideas. Such new ideas, then, may be embraced by the scientific community boosting one’s career, but may also be rejected hampering one’s career. Having less diverse mentors is expected to lead only to a moderate success (*few* mentees later on) consistent with a pattern of incremental innovations of established lines of thinking.

In the following, we elaborate on our genealogical approach (section 2) and then empirically analyze a large-scale mentor-mentee database descriptively (section 3) and with regression models (section 4). We reflect on our results in the light of theories about novelty and formulate some implications for Ph.D. training within universities (section 5).

## 2 A genealogical framework

In the biological sphere, genealogy entails the reproduction of genetic material by parents in their offspring. Likewise, in the cultural sphere, cultural items are being replicated from person to person through training. For example, in business studies, it has been found that the survival rate of a spinoff firm is correlated with the survival rate of the parent firm, which is explained by the knowledge and skills that the founder acquired while working for the parent firm [16–18]. In science, knowledge transfer between mentor and mentees is evident from the citation pattern of Ph.D. students, who are more likely to cite works by their supervisors than works by people with whom they do not have such a genealogical link [19].

Ph.D. training, however, does not only involve the reproduction of knowledge and skills as to qualify as an academic scientist, but also has as an explicit objective to produce new scientific knowledge. The future career of a Ph.D. student, then, does not depend so much on the faithful reproduction of the knowledge and skills of her or his supervisor(s), but much more on the ability to produce novelty in terms of new theoretical or empirical insights. For a genealogical approach to academic success, a key question is then where scientific novelty comes from. We propose two genealogical principles: ancestry size and ancestry diversity.

First, we argue that for Ph.D. students to become successful, it helps if they can build on the expertise of multiple supervisors rather than having to rely on just one. One can safely assume that a Ph.D. student with multiple supervisors will not receive the exact same knowledge inputs from each of the supervisors. Receiving more inputs means that one can create more new knowledge, following the view that more knowledge inputs allow for more re-combinational options [14, 15]. Following this view, the more supervisors a Ph.D. student has, the more knowledge inputs they will receive, and the more knowledge output they will produce.

Following the genealogical logic, this argument extends to multiple generations. That is, as mentors themselves have been a mentee in the past, a scientist is not just influenced by its own mentor(s), but also, indirectly, from the mentor(s) of mentor(s), and so on. Mentoring influences thus are not only direct, but can also be indirect via mentors of mentors. In principle, genealogical relationships can be traced back *ab initio* in a family tree. A genealogical approach thus allows one to consider the whole ancestry of a mentee, rather than only focusing on the mentor-mentee dyad. Having more mentors–be them direct or indirect–is thus assumed to lead to more knowledge inputs, which in turn leads to more academic success. This leads us to the first hypothesis:

*Hypothesis 1*: *A large ancestry increases the likelihood of academic success*.

Our second genealogical argument reasons from the diversity of inputs. While more knowledge inputs are expected to increase academic success, the nature of knowledge inputs is also expected to matter. Knowledge inputs from multiple supervisors are arguably always different to some extent, but the difference between inputs may vary from very similar to very dissimilar. That is, the mentoring team may be more or less diverse in terms of the knowledge inputs they provide. The diversity of the mentoring team is a function of the ‘cognitive distance’ between the mentors, where cognitive distance can be understood as the extent to which two individuals have different knowledge [20]. In the case of Ph.D. supervisors, the cognitive distance between two supervisors may reflect their disciplinary backgrounds, theoretical orientations, methodological skills, etc. For example, one can consider the knowledge of a physics mentor and a psychology mentor to be more diverse than, say, the knowledge of a computer scientist and an applied mathematician.

Dissimilar inputs from a diverse set of mentors can help the mentee to develop radically new ideas by recombining these inputs resources [21, 22]. Reversely, when mentors are similar, the resulting ideas produced by the mentee are more likely to resemble the inputs recombined and, accordingly, will be less radical. However, we argue that for a mentee it will be harder to successfully combine dissimilar knowledge inputs than similar knowledge inputs, as dissimilar inputs refer to different, and often incompatible assumptions, theories and methodologies. Successfully recombining dissimilar inputs would require many more mobilized resources and skills and more creativity. Moreover, the high degree of novelty of the knowledge produced may not be accepted by the academic community, as it deviates from established lines of thinking [23]. By contrast, similar knowledge inputs are easier to combine into incrementally new knowledge, since these are based on the same background knowledge, skills, and resources [21, 22]. Hence, while dissimilar inputs may provide opportunities for radically new ideas, it also constitutes a riskier context in which mentees may fail to advance science in a meaningful and accepted manner. This leads us to the second hypothesis:

*Hypothesis 2*: *A diverse ancestry increases the likelihood of academic success*, *and of academic failure*.

## 3 Methods

### 3.1 A genealogical network approach

In demographic studies, genealogies are used to study ancestral lineages in a population [24]. Genealogies can be represented as networks called pedigrees, where edges link parents to offspring. Nodes, in these networks, represent individuals, while edges represent historic relations between nodes. In-degree refer to an individual’s number of direct ancestors, and out-degree to the number of direct descendants. Edges are directed away from the common ancestor at the root of the network. Genealogical networks are acyclic, meaning that they cannot contain directed cycles [25].

**Fig 1:**
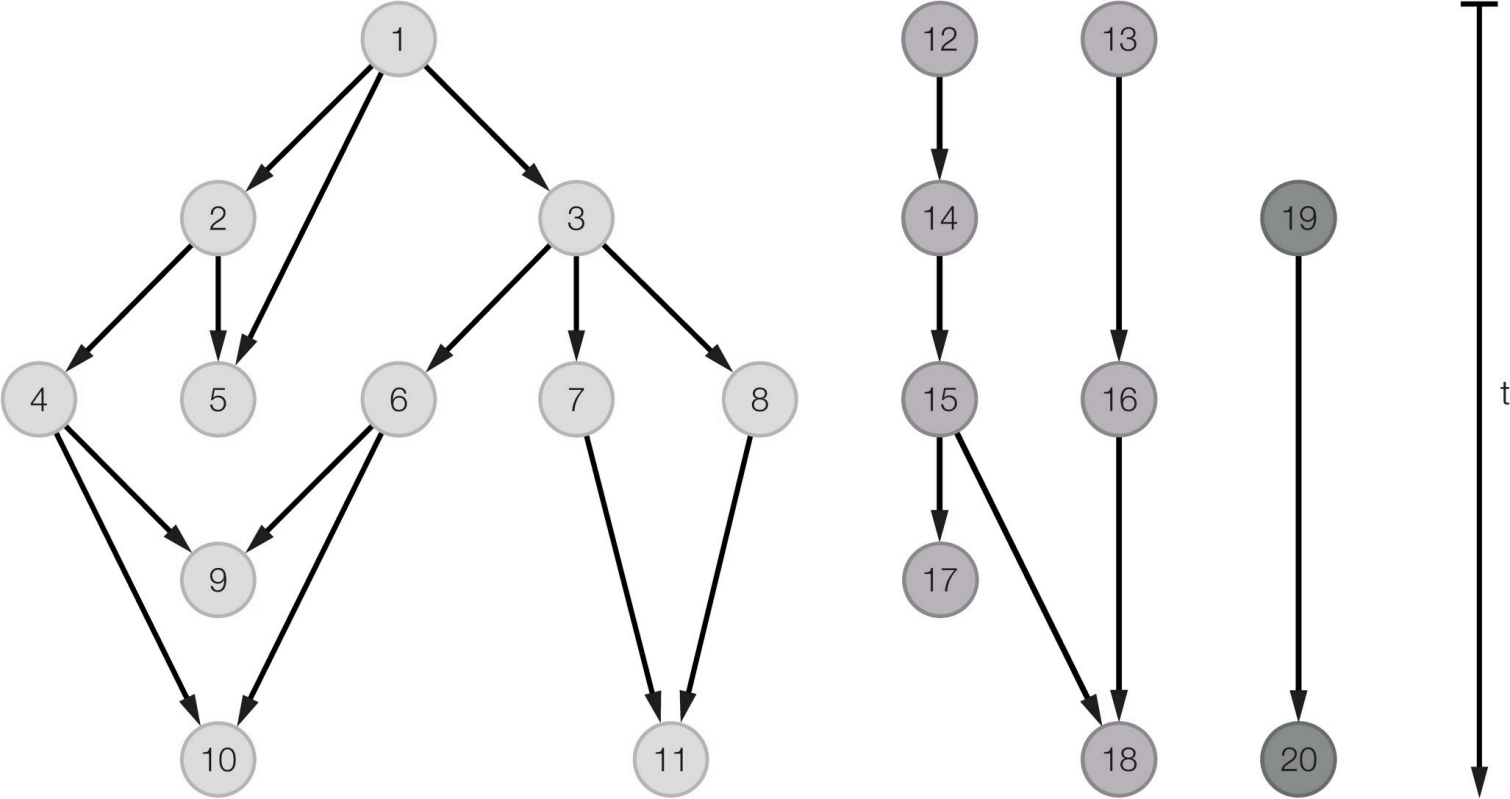
Example of a subnetwork of a genealogy of scientists.

In this paper we use a genealogical network to represent mentor-mentee relationships, where the nodes represent individual scientists and edges represent supervisory relationships between mentors and mentees [7, 11, 13]. Accordingly, we can specify mentor-mentee relationships and speak of ancestors and descendants in an academic genealogy. Fig 1 presents a hypothetical subnetwork with 20 individuals.

Network structures can be described at the micro-level of an individual scientist and at the macro-level of the entire network. At the *micro*-level, in-degree denotes the number of mentors of an individual and out-degree denotes the number of mentees of an individual. Furthermore, the number of co-mentees can be expressed by the number of individuals with whom an individual shares a mentor. In this example, individual 3 is the *mentor* of individual 6, while individuals 9 and 10 are *mentees* of individual 6. Individual 6, then, has an in-degree of 1 and an out-degree of 2. Besides individual 3, individual 1 also belongs to the *ancestors* of individual 6, as individual 1 is a *second-order mentor* of individual 6. And, individual 1 is a *third-order mentor* of individuals 9, 10 and 11.

**Fig 2:**
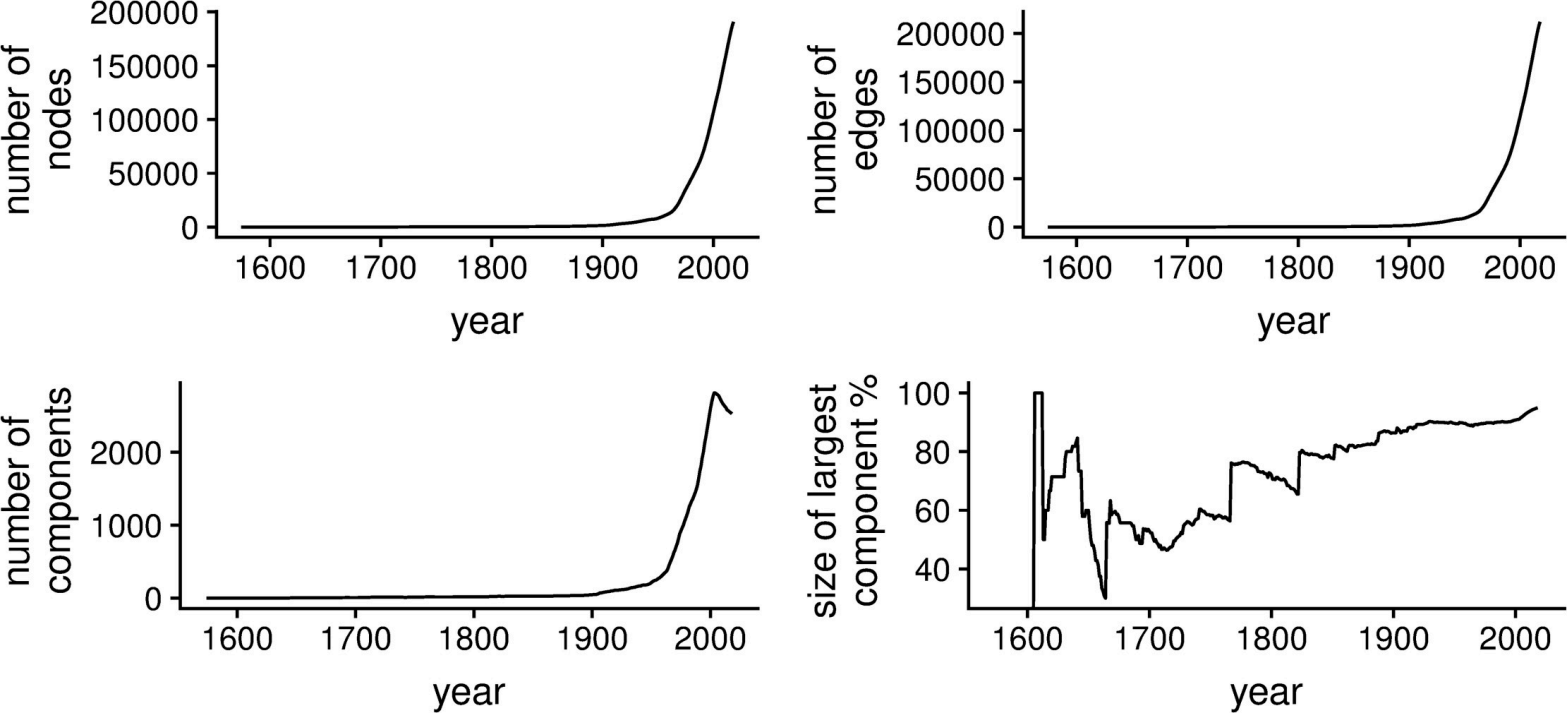
Network characteristics. Number of nodes (scientists with a Ph.D. degree) (upper left), number of edges (mentor-mentee relationships) (upper right), number of components (bottom left), and relative size of the largest component (bottom right).

At the *macro*-level, the longest path of directed edges in the network, called the network’s diameter, reflects the total number of generations [25]. In Fig 2, the longest path of directed edges is 3 (from individual 1 to individuals 9, 10 and 11). A diameter of 3 implies that there are four generations of scientists. Another macro-level property is the number of components and their size. In the example, we have three components, with the largest component containing 11 nodes, the second largest component 7 nodes and the smallest component only 2 nodes. Over time, components may merge into one: for example, if individuals 11 and 18 supervise a new node, then their respective components will merge into one larger component with size 11+7+1 = 19.

### 3.2 Data

To analyse the determinants of scientists’ academic success, we use a large-scale genealogical network of scientists. Our genealogical data is drawn from the Mathematics Genealogy Project, an online database developed as a historical record of scientists with a Ph.D. in mathematics to keep track of mentor-mentee relationships [26]. For our analysis, we have obtained the data through recursive queries to the Mathematics Genealogy Project using R and the R-package ‘rvest’ [27]. Queries ran between the 27th of June 2018 and the 10th of July 2018.

The project has been a source of inspiration for similar data collection efforts in other scientific disciplines [28, 29]. Despite its crowdsourced nature, this database is recognized as one of the most complete historical Ph.D. records in the field of Mathematics [12, 30]. Over time, it has come to include other scientific fields that are associated with mathematics, primarily, physics and chemistry. The case of mathematics, physics and chemistry is especially useful for studying the evolution of scientific knowledge, because these fields are less constrained by geographical and political interests, language barriers and access to materials than social sciences, humanities, and engineering [31]. Therefore, these constraints that typically affect the evolution of scientific knowledge are somewhat less relevant for mathematics, physics and chemistry than for other disciplines.

Each scientist is registered by a unique ID. The project also records the institute and country where the Ph.D. degree was obtained, the year in which the degree was obtained, and, often, the title and subject classification of the thesis. No other personal information is provided in the dataset. The earliest observation in the network is from the year 1574, the latest from 2018. The total network contains 196,667 nodes and 288,147 edges, distributed over 24 generations. Fig 2 presents the development of the network between 1574 and 2018. We see that the total amount of Ph.D. degrees awarded annually has started to grow rapidly after 1945 (Fig 2, top left) and, correspondingly, the amount of mentor-mentee relationships (Fig 2, top right), and the number of components in the network (Fig 2, bottom left). The relative size of the largest component (Fig 2, bottom right) tends to increase, with sudden large jumps in the years 1766 and 1822 resulting from mergers of components. After that, the largest component covers the large majority of scientists.

**Fig 3:**
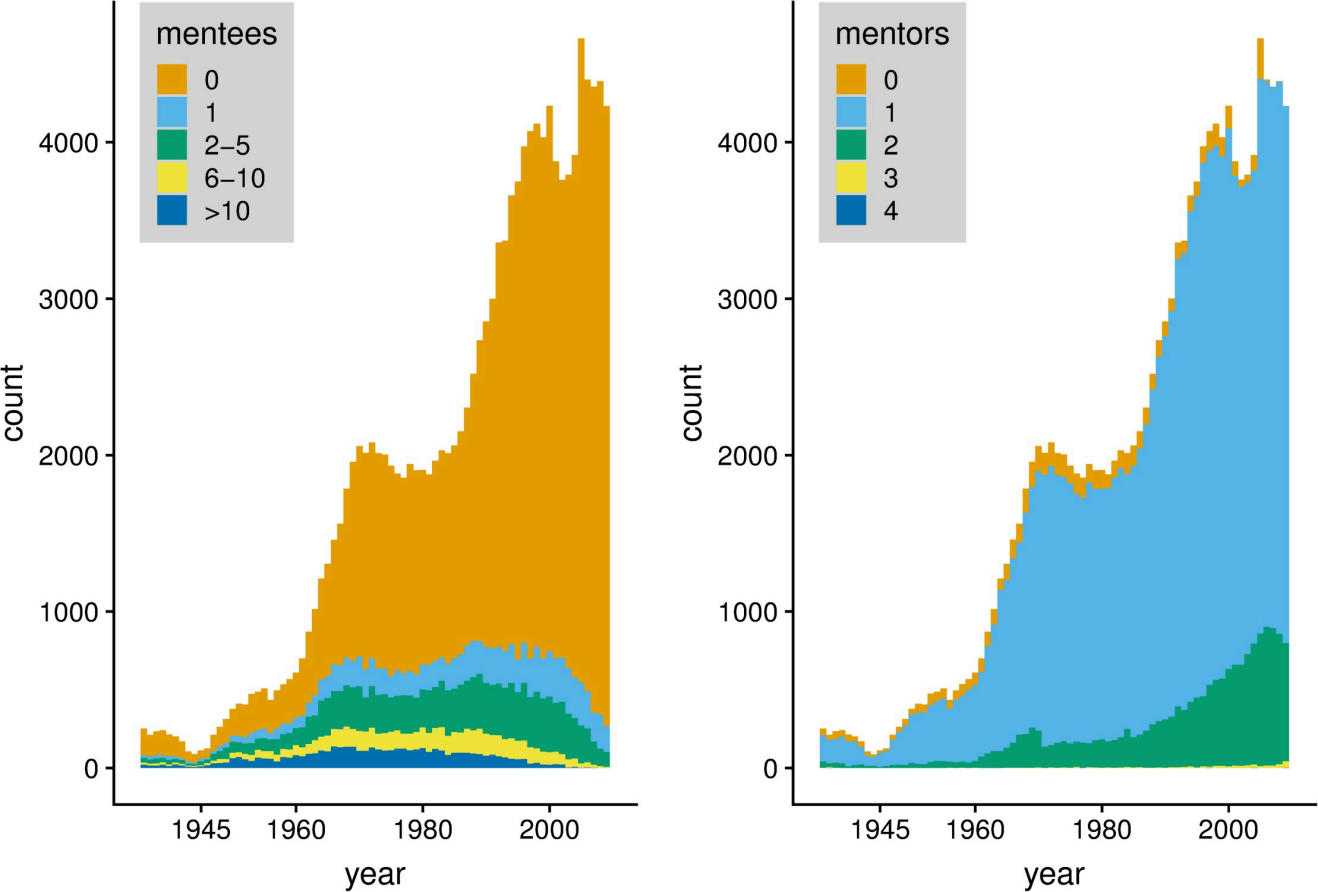
Count of scientists per year, per number of mentees (figure left) and mentors (figure right).

For our analysis, we limit the data set to scientists who obtained their Ph.D. after 1945. The reason holds that only after 1945, it became more common to have multiple formal supervisors (Fig 3, right). Furthermore, we do not include scientists who obtained their Ph.D. degree in 2000 or later, because there is a time lag in collecting mentees due to career progression (Fig 3, left).

Fig 3 shows the number of observed scientists in our data set per year, as well as the distribution of the number of mentors and mentees of our observed scientists. Scientists have, on average, 1.818 mentees and 1.054 mentors. This discrepancy is due to the sharp increase in Ph.D. graduates in the last years. In the left figure in Fig 3, we can see that many Ph.D. graduates do not become a mentor themselves, as the majority of scientists have zero mentees. The fraction of scientists with zero mentees increases over time. The distribution is also very skewed. In the right figure in Fig 3 we can see that having zero reported mentors is extremely rare. Such observations may either refer to scientists who obtained a professorship without a Ph.D. degree, or whose supervisor’s name is missing in the dataset. Hence, to the extent that scientists without mentors would signal the inaccuracy of the dataset, the rareness of it can be seen as a sign of the quality of the dataset. Fig 3 further shows that the majority of Ph.D. students have one mentor, but a considerable share of Ph.D. students has two mentors. Only 184 Ph.D. students in our data set have 3 mentors, and 13 individuals have 4 mentors. An in-degree higher than four does not occur in this dataset. Note that in some rare cases, individuals may have more than one mentor because they wrote multiple Ph.D. theses with different mentors, rather than having multiple mentors for one thesis.

### 3.3 Model

The phenomenon we aim to explain is academic success as measured by a count of a scientist’s *mentees*, that is, the out-degree. The large number of scientists with zero mentees reflects that many scientists who earned a Ph.D. degree did not continue their career in academia and, consequently, did not mentor any mentees (Fig 3, left). This can be because they were not susceptible to an academic position after their Ph.D., but also because they may have found job opportunities in other industries more attractive. Others may have continued in academia but never successfully attracted mentees, particularly if working in smaller and less reputed universities where Ph.D. programs are underdeveloped or in academic institutions others than universities. While such scientists may have achieved considerable success in other ways, they have not been able to transfer their scientific ideas to new generations of scientists by training Ph.D. students.

Given the excessive number of zeroes in the dependent variable, we apply a zero-inflated negative binomial (ZINB) regression model. Here, we assume that having zero mentees can be the outcome of two separate processes [7, 32]: first, one’s susceptibility to acquiring an academic professor position (as modelled in the logit part of our analysis, explaining zero mentees), and second, ability to attract and successfully supervise mentees as professor (as modelled in the negative binomial part of our analysis, explaining the number of mentees). The negative binomial part of our analysis thus estimates the expected number of mentees, conditional on being able to acquire a professor position.

Following hypothesis 1 (‘*A large ancestry increases the likelihood of academic success’*), we first test the effect of ancestry size on the number of mentees. We do this for the full set of scientists in the dataset (N = 95,502). Here, we distinguish between different generations of mentors (*first-order mentors*, *second-order mentors* and *third-order mentors*), expecting that the effect of more distant generations will become progressively smaller. For each scientist in the network, we thus compute the following characteristics that, together, represent ancestry size: (a) number of first-order mentors–corresponding to in-degree; (b) number of second-order mentors–corresponding to the sum of an individual’s mentors’ in-degrees; and (c) number of third-order mentors–corresponding to the sum of second-order mentors’ in-degrees. In order to prevent multicollinearity, our variables for *second-* and *third*-*order mentors* are normalized, meaning that they represent the mean number of second-order mentors per first-order mentor and the mean number of third-order mentors per second-order mentor. For example, in Fig 1, individual 10 has two mentors who, themselves, both have one mentor. So, individual 10 has one second-order mentor per first order mentor. And, individual 10 has one third-order mentor per second-order mentor, because both her second-order mentors have one mentor (individual 1).

Table 1 provides the descriptive statistics of the genealogical variables used in our model. We can observe that the mean number of first-, second- and third-order mentors is close to one. Few scientists have a high number of second- or third-order mentors.

**Table 1:** Descriptive statistics for genealogical variables.

Variable	N	Mean	St. Dev.	Min	Pctl(25)	Pctl(75)	Max
mentees	95,502	1.818	5.014	0	0	1	144
first-order mentors	95,502	1.054	0.407	0	1	1	4
second-order mentors (mean)	95,502	0.973	0.510	0	1	1	4
third-order mentors (mean)	95,502	0.933	0.553	0	1	1	4
ancestry diversity	9,296	0.770	0.354	0	0.8	1	1
mentees of first-order mentors (mean)	95,502	17.520	16.290	1	6	23	144

Turning to hypothesis 2 (*‘A diverse ancestry increases the likelihood of academic success*, *and of academic failure’*), we need to operationalize exceptional success and failure as well as the diversity of one’s ancestry. Here, academic success again means that a scientist has many mentees later on (as measured by the count in the negative binomial part in the ZINB-model), while academic failure here means that a scientist will have no mentees at all later on (as measured as zero mentees in the logit part of the ZINB-model).

*Ancestry diversity* is derived from the network distance between two mentors, which is the shortest path length between two mentors towards a common ancestor ‘upwards’ in the genealogy. For an example, consider again Fig 1. Individuals 4 and 6 have a network distance of 4, while individuals 7 and 8 have a network distance of 2. Network distance thus measures the cognitive distance between mentors *prior* to their joint mentoring of the Ph.D. student in question. A particular case is when two mentors come from different components in the network, such as individuals 15 and 16 in our example. Since such mentors have no common ancestor, they have an infinite network distance. To express *ancestry diversity* while taking into account infinite values for network distance, diversity is defined as one minus the inverse of network distance [23]. The value of *ancestry diversity* thus takes on the value of 1 if two mentors belong to two different components in the network and 0 when two mentors are the closest, that is, when one mentor supervised the other.

Ancestry diversity can only be applied to the minority of mentees who have more than one mentor. Among them, the very large majority (98%) has two mentors (see Fig 3 (right)). We test the second hypothesis using only the subset of mentees with two mentors (N = 9,296). From Table 1, we can learn that among the 9,296 Ph.D. students with two mentors, the mean *ancestry diversity* is 0.770. Also note that an *ancestry diversity* of 1 is not uncommon: 43.6% of Ph.D. students with two mentors have an infinite network distance. Besides infinite distance, the largest network distance that we observe in our dataset is 29, yielding an *ancestry diversity* value of close to 1 (to be precise: 1 – (1/29) = 0.965).

In all regression analyses, we include a number of control variables that are likely to affect the number of mentees of a scientist and may confound the effect of the ancestry size and ancestry diversity, if neglected. First, there must be scientists in our data set who did not have any mentees, not because they did not have the potential to acquire them, but because they considered external job opportunities more attractive. We cannot consider these scientists as having failed academically, because they may not have had the ambition to pursue an academic career. This is particularly likely for scientists with a Ph.D. from engineering universities and medical universities, for whom there may be many attractive career options outside of academia and for whom the prospect of an academic career may not be their main consideration to get a Ph.D. degree in the first place [33]. In this context, a recent study estimated that about half of Ph.D. students in Europe consider their training as relevant to a wide range of careers [34]. We therefore include a dummy variable *technical & medical university*, referring to Ph.D. student who received their degree from technical university or medical school. Following Frenken, Heimeriks and Hoekman [35], we classified universities as technical and based on the university’s name and, for those for which the name does not specify any such specialization, the university’s home page was checked for mentioning a technical or medical specialization.

Second, as we are analysing a network, there may be interdependencies between individual scientists’ number of mentees, and the number of mentees of their mentor(s) in the network. This is even likely, given our genealogical network, as talented people may be drawn to talented supervisors. Then, it is likely that mentors with a large number of mentees have had mentors who, themselves, have had a large number of mentees [5, 16]. Therefore, we include the mean number of mentees of an individual’s first-order mentors, as a lagged dependent variable, which corresponds to the mean academic success of an individual’s mentors. Note that, as we see in Table 1, the mean number of mentees of first-order mentors is higher than the overall mean number of mentees, because the overall number of mentees includes many scientists with zero mentees, while the number of mentees of first-order mentees logically does not.

Finally, the number of mentees being trained has steadily risen over time (see Fig 3). We therefore include dummies for each *decade* from 1940s until the 1990s (with the 1940s being the reference category).

## 4 Results

### 4.1 Ancestry size and academic success

In Table 2, we present the results of four zero-inflated negative binomial regression models. We model the count variable *mentees* using measures of ancestry size as independent variables. In models (1)-(3) we add the three ancestry size variables one by one, while we base our interpretation on model version (3), which includes all variables We control for *decade* and *technical & medical university* in all models.

**Table 2:** Zero-inflated negative binomial regression coefficients for ancestry size (dependent variable: Mentees).

	(1)		(2)		(3)	
	logit	count	logit	count	logit	count
first-order mentors	-0.323***	0.254***	-0.327***	0.255***	-0.322***	0.252***
	(0.037)	(0.026)	(0.037)	(0.026)	(0.037)	(0.026)
second-order mentors			-0.057*	0.064**	-0.044	0.039^
			(0.027)	(0.021)	(0.028)	(0.021)
third-order mentors					-0.035	0.075***
					(0.025)	(0.019)
mentees of first-order mentors	0.009***	0.010***	0.009***	0.009***	0.009***	0.009***
	(0.001)	(0.001)	(0.001)	(0.001)	(0.001)	(0.001)
technical & medical university	0.046^	0.127***	0.046^	0.131***	0.046^	0.126***
	(0.036)	(0.029)	(0.036)	(0.029)	(0.036)	(0.029)
decade: 1950s	0.176	-0.068	0.176	-0.067	0.178	-0.067
	(0.163)	(0.068)	(0.163)	(0.068)	(0.162)	(0.068)
decade: 1960s	0.847***	-0.260***	0.847***	-0.259***	0.846***	-0.253***
	(0.152)	(0.064)	(0.152)	(0.064)	(0.151)	(0.064)
decade: 1970s	1.415***	-0.300***	1.414***	-0.294***	1.411***	-0.289***
	(0.152)	(0.064)	(0.152)	(0.064)	(0.151)	(0.064)
decade: 1980s	1.214***	-0.595***	1.211***	-0.592***	1.207***	-0.582***
	(0.152)	(0.063)	(0.151)	(0.063)	(0.150)	(0.063)
decade: 1990s	1.835***	-1.062***	1.831***	-1.061***	1.826***	-1.048***
	(0.152)	(0.063)	(0.151)	(0.063)	(0.151)	(0.063)
constant	-1.226***	1.290***	-1.214***	1.288***	-1.140***	1.176***
	(0.163)	(0.069)	(0.282)	(0.075)	(0.166)	(0.074)
observations	95,502		95,502		95,502	
log likelihood	-123,855		-123,871		-123,829	
Nagelkerke R2	0.250		0.250		0.251	

*Note*:

^p<0.1

*p<0.05

**p<0.01

***p<0.001. Only scientists who obtained their Ph.D. degree between 1945 and 2000 are included in the regression (N = 95,502). Decade 1940s is the reference category.

In the logit part of our models, we find that having more supervisors (*first-order mentors*) decreases the likelihood of having zero mentees (that is, each extra mentor decreases the likelihood of zero mentees with a factor e^-0.322^ = 0.725). This shows that those with multiple supervisors are much more likely to ultimately be in a position to mentor others. We do not, however, find a robust effect of *second-* and *third*-*order mentors* on having zero mentees.

We further find that scientists with a Ph.D. degree from an engineering university or medical university are more likely to have zero mentees (odds ratio (OR) = e^0.046^ = 1.047). We can interpret this finding as an indication that such scientists are indeed more likely to face attractive career opportunities in industry or the hospital sector, respectively, leaving them less susceptible to an academic career. However, the effect is neither very high nor very significant statistically. Interestingly, we also find a positive and significant effect of *mentees of first-order mentors* in the logit part of model (3), suggesting that other descendants of one’s ancestors increase the likelihood of not acquiring a mentoring position. This might be because such scientists may compete for the same positions [36, 37]. The decade dummies finally suggest that, over time, the likelihood of Ph.D. students to become a mentor themselves decreases gradually.

We find that in the negative binomial part of model (3), *first-order mentors* positively affect the expected value of *mentees*. As such, the number of direct mentors positively affects academic success. There is also a (much smaller) positive and significant effect of *second-* and *third*-*order mentors* on academic success. *Technical & medical university* and *mentees of first-order mentors* both positively affect the expected number of mentees of an individual. Finally, we find that the potential of academic success gradually decreases over time, compared to the 1940s, as shown.in the count part of our model (3),

In sum, we find support for our first hypothesis (‘*Scientists who have had more direct and indirect mentors*, *will experience more academic success’*). Controlling for technical and medical universities, decade, and mentor success, we find that having more mentors decreases one’s likelihood of having zero mentees, and increases one’s expected number of mentees. This effect is particularly clear for direct mentors, and weaker for indirect mentors.

### 4.2 Ancestry diversity and academic success

Table 3 presents the results for ancestry diversity of our zero-inflated negative binomial regression models based on a subset of our data consisting of 9,296 scientists who had two mentors. In model (4), we control again for decade, technical and medical universities, and mentor success in our models. In the final model (5), we also control for ancestry size effects of second- and third-order mentors, to see if results from model (3) in Table 2 are robust. We do not control for the number of first-order mentors, as this number if always 2 in the subset we consider here.

**Table 3:** Zero-inflated negative binomial regression coefficients for ancestry diversity (dependent variable: Mentees).

	(4)		(5)	
	logit	count	logit	count
ancestry diversity	0.136*	0.092***	0.127*	0.071***
	(0.063)	(0.019)	(0.065)	(0.020)
second-order mentors			0.057	0.103***
			(0.056)	(0.017)
third-order mentors			0.054	0.095***
			(0.053)	(0.016)
mentees of first-order mentors	0.007***	0.009***	0.006**	0.008***
	(0.002)	(0.001)	(0.002)	(0.001)
technical & medical university	0.019	0.092***	0.015	0.083***
	(0.067)	(0.020)	(0.067)	(0.020)
decade: 1950s	-0.029	-0.196***	-0.031	-0.200***
	(0.249)	(0.044)	(0.249)	(0.044)
decade: 1960s	0.611**	-0.459***	0.606**	-0.466***
	(0.226)	(0.041)	(0.226)	(0.041)
decade: 1970s	0.917***	-0.329***	0.918***	-0.325***
	(0.226)	(0.041)	(0.226)	(0.041)
decade: 1980s	1.072***	-0.596***	1.077***	-0.587***
	(0.225)	(0.041)	(0.225)	(0.041)
decade: 1990s	1.598***	-0.994***	1.604***	-0.983***
	(0.223)	(0.042)	(0.223)	(0.042)
constant	-0.701**	2.263***	-0.795***	2.101***
	(0.229)	(0.043)	(0.237)	(0.047)
observations	9,296		9,296	
log likelihood	-19,965		-19,924	
Nagelkerke R2	0.249		0.257	

*Note*:

^p<0.1

*p<0.05

**p<0.01

***p<0.001. Only scientists with more than two mentors in time period 1945–2000 are included in the regression (N = 9,296). Decade 1940s is the reference category.

In model (5), we find that *ancestry diversity* significantly affects *mentees*. We find that a one-unit increase in *ancestry diversity* increases the expected number of mentees conditional on acquiring an academic position (with a factor e^0.071^ = 1.074)), but also increases the likelihood of having zero mentees (with a factor e^0.127^ = 1.135), as hypothesized. Including ancestry size variables in model (5) does not significantly change the effect of *ancestry diversity* on *mentees*, which shows that our results in Table 3 are consistent with those found in model (3) in Table 2. These findings shed a new light on mentor diversity compared to a previous study (7) where mentor diversity was shown to increase the likelihood of acquiring an academic position, and to have no effect on the number of mentees.

In summary, we find that the effect of *ancestry diversity* in both the count models and the logit models is positive and significant, thereby providing evidence in support of our second hypothesis (‘*A diverse ancestry increases the likelihood of academic success*, *and of academic failure’)*.

## 5 Discussion

We developed a genealogical framework to explain the academic success of Ph.D. students later on in their careers. We focused on the size and diversity of Ph.D. student’s ancestry, and reasoned that one’s ancestors provide valuable knowledge inputs that one can recombine and pass on to new generations of Ph.D. students. As such, we argue that scientists who acquire many mentees to pass on their knowledge can be considered academically more successful than scientists who do not, at least from a genealogical perspective.

Our results suggest that the more supervisors are involved with a Ph.D. student who is susceptible to an academic career, the more academically successful this student will be later on. That is, individuals with more mentors later on generate more mentees, suggesting that, in a general sense, Ph.D. students benefit from any knowledge input. Looking more closely at ancestry diversity, we find, different from a previous study (7), that network distance between an individual’s mentors–as a proxy for cognitive distance–makes both success and failure more likely. Cognitive distance between supervisors may reflect different disciplinary backgrounds, theoretical orientations, and methodological skills, or in short, knowledge diversity. We hypothesized that such a diversity of inputs provides opportunities for radically new ideas, but are poses challenges how recombine inputs in a meaningful way. Our empirical findings provide support for this hypothesis as ancestry diversity is shown to more often lead to extreme success and failure. More generally, our findings indicate that to understand academic success by looking at mentorship, it is important to take all mentors into account rather than only one as has been common in previous studies (8–10).

Practically, our findings have implications for the effective organization of Ph.D. training. In general, adding another supervisor to a team pays off. And, if two supervisors are cognitively diverse, one may benefit from the opportunities to make very new combinations of existing knowledge. However, such contexts are also more prone to failure. In other words, one can think of having diverse supervisors as a ‘high-risk, high-gain’ strategy for PhD students. This suggests that if projects are set-up with diverse supervisors, it may be worthwhile to foresee a back-up plan, in case progress is slow and collaboration turns out to be less fruitful than hoped for. Given the institutional differences across countries and disciplines regarding Ph.D. supervision, the organization and coordination of multiple supervisors can take on different forms. For example, in some contexts a Ph.D. student is part of a (externally funded) research project, with co-supervisors being involved from the start. Then, supervisory roles and responsibilities can be largely defined ex ante. In other contexts, aspiring Ph.D. students submit their own research proposal to a graduate school and may select their own supervisor. Here, the organizational question becomes how to match students and supervisors and under what conditions a student is allocated multiple supervisors.

Our study illustrates the power of applying a genealogical approach to the analysis of human creativity, innovation and success. It complements the already extensive literature on team collaboration and innovation [19, 38, 39], which has thus far mainly reasoned from the perspective of senior scientists rather than the Ph.D. students. In evolutionary terms, collaboration assumes horizontal transmission among team members, to be analyzed as an undirected network, while genealogy looks explicitly at vertical transmission, to be analyzed as a directed network. A next step is to combine both perspectives in a single framework, for example, by analyzing not just mentors of mentees, but also the co-location of mentees at the same moment in time. Such peers may cross-fertilize their ideas, yet may also compete for similar jobs in the further stages of their career. Indeed, our results support the idea that mentors with many mentees bring about successful mentees [5], but also that having a productive mentor may be a source of competition [36].

Our study also contributes to the ‘proximity’ literature [22], by introducing an alternative understanding of the concept of cognitive distance [40]. It has been argued that engaging with cognitively distant partners in collaboration is a balancing act between individuals’ ability to understand each other (supported by cognitive proximity), and their potential to learn from one another (supported by cognitive distance). This results in an ‘optimal cognitive distance’, above and below which the difficulties may outweigh the benefits. That is, assuming risk aversity, the optimal strategy is to have multiple mentors with only moderate cognitive distance. Our results further suggest that ideas that arise under circumstances of high cognitive distance may often fail, but may also lead to a very productive career later on. This means that the ‘optimum’ is not a universal value, but depends on the risk preferences of actors involved: if one engages in a risky strategy and aims for very novel knowledge, the optimal cognitive distance is higher than if one aim for more incremental knowledge. The institutional and disciplinary contexts may also matter. Some research contexts are more liberal and open allowing students to be mentored by a diverse set of mentors, while other research contexts may be more disciplinary and focused, following certain standards and traditions akin to a research school [41].

There are several limitations to our work. First, there may be a self-selection bias, particularly in terms of ancestry size. It might be the case that skilled Ph.D. students are more likely to work with a larger set of mentors, possibly because they have more specific mentoring demands. However, given that we control for mentor success, we believe that the self-selection effect is small.

Secondly, our success variable, number of mentees, is defined genealogically rather than in terms of academic quality or impact. Indeed, the number of mentees that a scientist supervises is not a direct measure of the quality of research. Moreover, not all skilled scientists aspire to become a mentor in the first place. Alternative indicators of quality could be prizes awarded or number of citations received. We have chosen the number of mentees as a dependent variable not only to showcase the power of genealogical thinking but also for reasons of data availability, given that alternative indicators are hard to collect for long periods of time. And, we do consider the number of mentees as a proper proxy of quality as it has been shown to correlate with well-known indices of academic quality [5].

Third, our reliance on the Mathematics Genealogy Project (MPG) database comes with some inherent limitations. The data are crowdsourced but curated by the MPG team. This ensures a high quality of the data; yet, the crowdsourced nature of the raw data implies that the database itself is incomplete in that some scientists are missing. There is only one alternative world-wide database called academictree.org [7, 42], which is similarly crowdsourced but not curated [7], and one official U.S. national database called the ProQuest PhD Dissertation & Thesis Databank [8]. Interestingly, these databases by now cover many disciplines. Exploiting such databases in the future would allow one to test our hypotheses across disciplines. Our use of the MPG database also implied that we abstracted from personal characteristics of scientists as such information is not available in the database (nor in academictree.org). We deemed the manual collection of personal data to be infeasible given the high number of observations in the dataset, indicative of a trade-off between a high number of observations and low number of independent variables in these types of analyses.

Finally, our work focused mainly on the context of mathematics, physics and chemistry. We realize that this limits the generalizability of our study. However, in terms of our theoretical understanding of knowledge creation as a process of recombination, we believe that our framework may well apply to other scientific disciplines as to other fields of human creativity [16–18]. We hope that our study will inspire colleagues to start collecting genealogical data on other contexts as well, both within and outside academia.
